# Qualitative Profiling and Quantitative Analysis of Major Constituents in Jinmu-tang by UHPLC-Q-Orbitrap-MS and UPLC-TQ-MS/MS

**DOI:** 10.3390/molecules27227887

**Published:** 2022-11-15

**Authors:** Seol Jang, Ami Lee, Youn-Hwan Hwang

**Affiliations:** 1 KM Convergence Research Division, Korea Institute of Oriental Medicine, Yuseong-daero 1672, Yuseong-gu, Daejeon 34054, Republic of Korea; 2 Korean Convergence Medicine Major KIOM, University of Science & Technology (UST), Daejeon 34054, Republic of Korea

**Keywords:** Jinmu-tang, traditional herbal medicine prescription, UHPLC-Q-Orbitrap-MS, UPLC-TQ-MS/MS, quality control

## Abstract

Jinmu-tang (JMT) is a traditional herbal medicine consisting of five herbal medicines: *Poria cocos* Wolf, *Paeonia lactiflora* Pallas, *Zingiber officinale* Roscoe, *Atractylodes japonica* Koidzumi, and *Aconitum carmichaeli* Debeaux. In this study, the JMT components were profiled using UHPLC-Q-Orbitrap-MS, and 23 compounds were identified and characterized. In addition, UPLC-TQ-MS/MS analysis was performed in the positive and negative ion modes of an electrospray ionization source for the simultaneous quantification of the identified compounds. The multiple reaction monitoring (MRM) method was established to increase the sensitivity of the quantitative analysis, and the method was verified through linearity, recovery, and precision. All analytes showed good linearity (R2 ≤ 0.9990). Moreover, the recovery and the relative standard deviation of precision were 86.19–114.62% and 0.20–8.00%, respectively. Using the established MRM analysis method, paeoniflorin was found to be the most abundant compound in JMT. In conclusion, these results provide information on the constituents of JMT and can be applied to quality control and evaluation.

## 1. Introduction

Traditional oriental medicines (TOM) and their preparations have been widely used for a long time in clinical practice for the treatment and prevention of various diseases in many Asian countries such as China, Korea, and Japan [1,2]. Jinmu-tang (JMT) is one of the classic prescriptions documented in the ‘Treatise on Febrile Diseases’, and consists of five single herbal medicinal: *Poria cocos* Wolf, *Paeonia lactiflora* Pallas, *Zingiber officinale* Roscoe, *Atractylodes japonica* Koidzumi, and *Aconitum carmichaeli* Debeaux [3]. In TOM, JMT has been used as a treatment for various kidney diseases because of its effectiveness in relieving symptoms caused by the deterioration of kidney functions [4]. Clinically, JMT improves kidney damage or function in glomerulonephritis, membranous nephropathy, and chronic renal failure [5,6,7]. Recent studies have revealed that JMT has various biological properties, such as anti-inflammatory, antioxidant, and anti-hyperlipidemia effects, following the pharmacological characteristics of every single herbal medicine [8,9,10].

TOM preparations consist of a variety of herbal medicines, but most select specific herbal medicines to manage and evaluate their quality. However, this does not accurately reflect the quality of the formulation, and the evaluation of efficacy determined by the pharmacological effects of various chemical components is also limited [1,11,12]. Currently, no studies have been reported on JMT quality control, so it is necessary to provide information on the constituents of JMT and establish an appropriate analysis method. According to previous studies, some components have been identified using high-performance liquid chromatography (HPLC) and gas chromatography-mass spectrometry (GC-MS) for quality evaluation of TOM preparations [13,14]. In particular, HPLC-MS is widely used in the analysis of TOM preparations because of its high sensitivity and resolution, and it is possible to identify multiple compounds in one analysis [15]. It also allows for systematic profiling of components, enabling convenient and rapid quality control [16]. JMT contains various components such as triterpenoids (e.g., pachymic acid) from *P. cocos* [17], monoterpenoids (e.g., paeoniflorin) from *P. lactiflora* [18], phenols (e.g., 6-gingerol), flavonoids (e.g., catechin) from *Z. officinale* [19,20], sesquiterpenoids (e.g., atractyloside A) from *A. japonica* [21], and alkaloids (e.g., fuziline) from *A. carmichaeli* [22].

However, there is little information about analytical methods for quality control of JMT. Therefore, in this study, the constituent compounds of JMT were identified using UHPLC-Q-Orbitrap-MS, and 23 components were characterized. In addition, the contents of 23 compounds were analyzed by validating and establishing a multiple reaction monitoring (MRM) method for the simultaneous quantification of the identified compounds.

## 2. Results and Discussion

### 2.1. Qualitative Analysis of Compounds in JMT by UHPLC-Q-Orbitrap-MS

**Figure 1:**
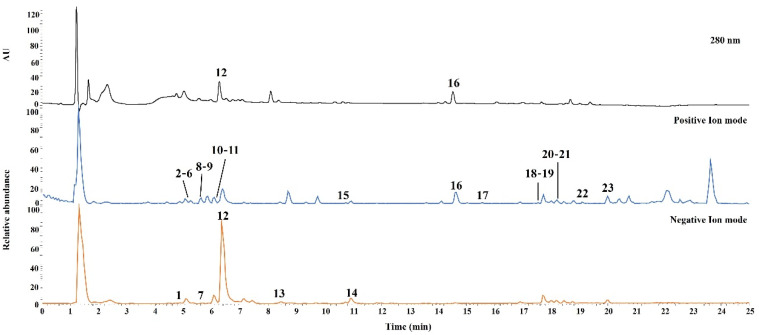
Representative base peak ion chromatograms of JMT extract by UHPLC-Q-Orbitrap-MS. The ID number of the various types of phytochemicals are listed in Table 1.

UHPLC-Q-Orbitrap-MS was performed for qualitative analysis of JMT, and the constituent chemical compounds were identified and characterized. Both the positive and negative ion modes were used to acquire the MS spectra. The UV and base peak chromatograms of JMT are shown in Figure 1.

In total, 23 compounds were identified and confirmed by comparing the retention times and mass spectra of authentic standard compounds. Detailed results for the characterized compounds, including retention times and MS data, are summarized in Table 1.

Of the chemical constituents identified in JMT in this study, six compounds (paeoniflorin, 6-gingerol, atractylenolide I, atractylenolide II, atractylenolide III, and pachymic acid) have been reported in previous studies [6,23].

### 2.2. Quantitative Analysis of Compounds in JMT by UPLC-TQ-MS/MS

**Figure 2:**
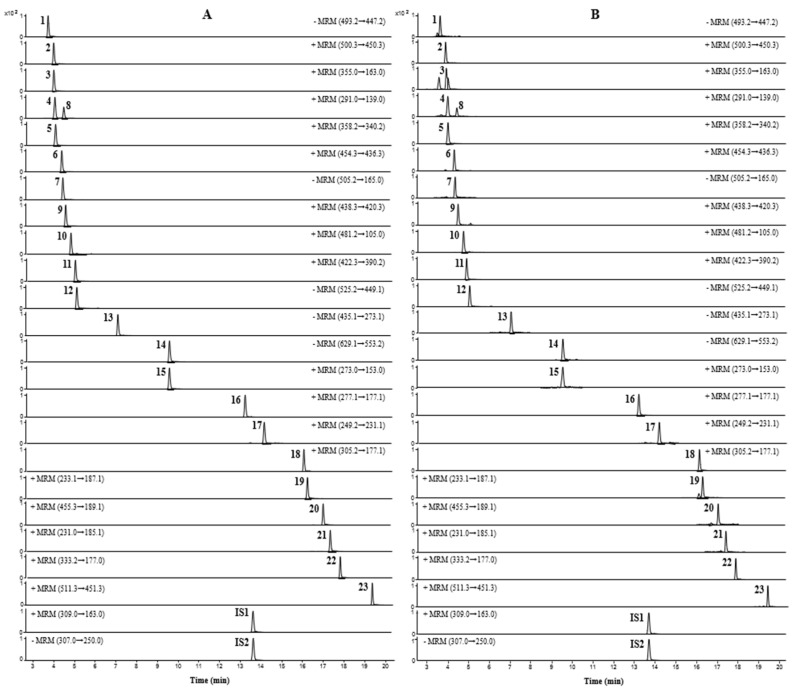
MRM chromatograms of mixed reference solution (**A**) and JMT extract (**B**).

UPLC-TQ-MS/MS was performed to quantify the 23 compounds of JMT, and all analytes were detected simultaneously within 20 min. To increase the sensitivity of the quantitative analysis, multiple reaction monitoring (MRM) was used and evaluated in both positive and negative ion modes. Five compounds (atractyloside A, paeonolide, paeoniflorin, phlorizin, and benzoylpaeoniflorin) were detected in negative ion mode, and the remaining 18 compounds were detected in positive ion mode. Optimized analysis conditions were established by identifying precursor ions for each analyte and selecting the product ions for MRM analysis. The MRM chromatograms obtained by applying the optimized analytical conditions are shown in Figure 2.

For aconine, *m*/*z* 450.3 was formed due to the loss of methanol (32 Da) and water (18 Da) molecules from the precursor ions [24]. For chlorogenic acid, one water molecule was lost from the dissociated caffeic acid moiety of the precursor molecule, and a product ion peak was detected at *m*/*z* 163.0 [25]. Catechin and epicatechin formed product ions at *m*/*z* 139.0, as a result of Retro-Diels-Alder (RDA) fragmentation (−152 Th) [26,27]. In napellonine, fuziline, and bullatine B, ions in the form of [M + H − H_2_O] were formed at *m*/*z* 340.2, 436.3, and 420.3, owing to the loss of H_2_O from the precursor ion in the form of [M + H]^+^ [28,29]. Paeonolide exhibited a characteristic ion at *m*/*z* 165.0 due to the loss of the adiglycosyl moiety [30], while phlorizin displayed a fragment at *m*/*z* 273.1 due to the loss of 162 Da from the precursor ion by deprotonated phloretin [31]. Albiflorin, a monoterpene glucoside, produced [M + H]^+^ ions at *m*/*z* 481.2 in positive ion mode. In contrast, paeoniflorin, an isomer of albiflorin, showed [M + COOH]^−^ ions at *m*/*z* 525.2 in the negative ion mode instead of the positive ion mode. In addition, benzoylpaeoniflorin showed fragmentation similar to paeoniflorin, and formed ions in the form of [M − H − HCOH]^−^; paeoniflorin and benzoylpaeoniflorin were observed at *m*/*z* 449.1 and *m*/*z* 553.2, respectively [32,33]. For talatisamine and naringenin, the product ion was formed in the positive ion mode from protonated molecular ions [M + H]^+^, and ions were generated at *m*/*z* 390.2 ([M + H − CH_3_OH]^+^) and *m*/*z* 153.0 ([M + H − C_8_H_8_O]^+^), respectively [34,35]. Furthermore, 6-gingerol, 8-gingerol, and 10-gingerol lost species at 100, 128, and 156 Da, respectively, due to the loss of the neutral alkyl moiety ([CH_3_(CH_2_)_n_CHO]) in the structure of [M + H − H_2_O]^+^. As a result, all three gingerols formed ions in the form of [M + H–H_2_O–C_6_H_12_O]^+^, [M + H–H_2_O–C_8_H_16_O]^+^, and [M + H–H_2_O–C_10_H_20_O]^+^ at *m*/*z* 177.1, respectively [36,37,38]. Among the three lactone components, atractylenolide I and II generated [M + H − H_2_O − CO]^+^ ions at *m*/*z* 185.1 and 187.1, respectively, as a result of the loss of H_2_O and CO groups from the precursor ions. In the case of atractylenolide III, [M + H − H_2_O]^+^ ion was formed at *m*/*z* 231.1 due to the loss of H_2_O molecules [32,39].

### 2.3. Method Validation of Qualitative Analysis

For the quantitative analysis of 23 compounds of JMT, linearity, recovery, precision, and accuracy were evaluated to verify the analytical method used in this study. The regression equations, correlation coefficients (R^2^), linear ranges, and LLOQ values for all the compounds are listed in Table 2.

The correlation coefficient, which indicates the linearity of the calibration curve, was greater than 0.9990 for all analytes, indicating good linearity. In addition, the LLOQ was in the range of 0.02–12.50 ng/mL for all compounds, which showed the sensitivity of the assay used in this study. The recovery was analyzed by adding mixed standard solutions of three different concentration levels to a known amount of the sample. The recoveries of the 23 compounds varied between 86.19% and 114.62%, and the relative standard deviations (RSD) values were found to be in the range of 0.38–5.80% (Table 3).

Precision and accuracy were validated by analyzing QC samples at three concentration levels. The intraday test was performed in six replicates within the same day, and the interday test was performed for three consecutive days. The intra- and interday precision calculated as RSD was less than 6.50% and 8.00% for all analytes, respectively, and the accuracy was within the range of 91.79–110.74 and 90.22–107.22%, respectively. Detailed data on precision and accuracy are shown in Table 4.

As described above, the analytical method used in this study was appropriately established based on the results of all validation parameters, suggesting that the simultaneous analysis of the 23 compounds of JMT can be performed accurately and efficiently.

### 2.4. Sample Analysis

Simultaneous quantitative analysis of the 23 compounds in JMT was performed by applying the validated UPLC-TQ-MS/MS method. The contents of all investigated compounds are summarized in Table 5 and were calculated by internal standard methods based on the respective calibration curves.

The UPLC-TQ-MS/MS MRM mode performed in this study was successfully applied to quantify the content of 23 compounds in JMT and all compounds eluted within 20 min. Among these, paeoniflorin, 6-gingerol, and albiflorin were found to be more abundant in JMT than in the other compounds. These three compounds are derived from *P. lactiflora* and *Z. officinale*, among the constituent herbal medicines of JMT, and these results are similar to those of previous reports on the ingredients of a single herbal medicine [18,40]. In addition, as in a previous report, the content of paeoniflorin was 25.694–27.876 mg/g in three batches of JMT, showing the highest content among the detection compounds [41].

## 3. Materials and Methods

### 3.1. Materials and Reagents

JMT consisted of five herbal medicines (*P. cocos*, *P. lactiflora*, *Z. officinale*, *A. japonica*, and *A. carmichaeli*), all of which were purchased from Kwangmyungdang Pharmaceutical (Ulsan, Korea). Each raw herbal medicine (specimens No. TDC-01 to TDC-05) was deposited in the KM Convergence Research Division, Korea Institute of Oriental Medicine. The reference standards for 23 components in JMT were purchased from Targetmol (Wellesley Hills, MA, USA) and ChemFaces (Wuhan, China). Warfarin, an internal standard, was obtained from Sigma-Aldrich (St. Louis, MO, USA), while MS-grade solvents (methanol, acetonitrile, water, and formic acid) were purchased from Thermo Fisher Scientific (Loughborough, UK).

### 3.2. Preparation of JMT

JMT, composed of five herbal medicines, was mixed in the ratio shown in Table 6, and distilled water (10-fold mass) was added and extracted under reflux at 100 °C for 3 h. The water extract was filtered, concentrated using a rotary evaporator under reduced pressure, and freeze-dried to prepare a powder sample (yield:22.0%).

### 3.3. Preparation of Standard and Sample Solutions

Stock solutions of the 23 compounds and warfarin (internal standard, IS) were prepared by diluting the reference standards with methanol. The stock solutions were then mixed, and a series of working solutions were obtained by further dilution with methanol. The mixed working solution was diluted to an appropriate concentration range for use in calibration curve construction, and the concentration of IS in each sample was kept consistent at 5.0 ng/mL. Quality control (QC) samples of the three concentration levels (high, medium, and low) used for method validation were prepared using the same method as the calibration samples. A sample solution of 20 mg JMT extract dissolved in methanol was subjected to ultrasonic extraction for 30 min. The extracted solution was then centrifuged at 12,500 rpm for 15 min and used for the analysis.

### 3.4. Qualitative Analysis

The chemical constituents of JMT were identified using ultra-performance liquid chromatography/quadrupole-Orbitrap mass spectrometry (UHPLC-Q-Orbitrap-MS) as previously reported [42]. A Dionex UltiMate 3000 system with a Thermo Q-Exactive mass spectrometer was used for analysis. Chromatographic analysis was performed using an Acquity BEH C18 column (100 × 2.1 mm, 1.7 μm) and a gradient mixture of 0.1% formic acid in water (A) and acetonitrile (B) was used for the mobile phase. The flow rate was 0.25 mL/min, and the sample injection volume was 3.0 μL. MS analysis was performed in positive and negative ionization switching modes using a Q-Exactive mass spectrometer, and MS spectra were obtained in full MS-ddMS2 mode. The other operating parameters were set as follows: spray voltage, 3.8 kV; capillary temperature, 320 °C; sheath gas pressure, 40 arbitrary units (au); auxiliary gas pressure, 10 au; resolution of MS scans, 70,000; resolution of MS/MS scans, 17,500; scan range, 100–1500 *m*/*z*; and normalized collision energy, 25 eV. The resulting data were acquired and analyzed using the Xcalibur v.3.0 and Tracefinder v.3.2 software (Thermo Fisher Scientific, Waltham, MA, USA).

### 3.5. Quantitative Analysis

Quantitative analysis of the 23 compounds in JMT was performed on an Agilent 1290 Infinity II system coupled to an Agilent 6495C triple quadrupole mass spectrometer with an electrospray ionization (ESI) source (Agilent Technologies, California, USA). The separation was achieved on an Acquity BEH C18 column (100 × 2.1 mm, 1.7 μm). The mobile phase consisted of 0.1% formic acid in water (A) and acetonitrile (B), and the gradient elution conditions were as follows:3% B (0–1 min), 3–15% B (1–2 min), 15–50% B (2–13 min), 50–100% B (13–20 min), 100% B (20–23 min), and 3% B (23.5–27.5 min). The column was maintained at 40 ℃, and the flow rate and injection volume were 0.25 mL/min and 3.0 μL, respectively. Mass spectra were acquired in positive or negative ion mode, and operating parameters were as follows: gas temperature, 130 ℃; drying gas flow, 11 L/min; nebulizer gas, 25 psi; sheath gas temperature, 400 ℃; sheath gas flow, 12 L/min; capillary voltage (positive mode), 3500 V; capillary voltage (negative mode), 3000 V; nozzle voltage (positive mode), 500 V; nozzle voltage (negative mode), 1500 V. The analysis of 23 compounds was performed using the multiple reaction monitoring (MRM) mode, and the detailed MRM conditions for each analyte are summarized in Table 7. The Agilent MassHunter workstation quantitative analysis software was used for all the MS data acquisition and processing.

### 3.6. Method Validation of Quantitative Analysis

The analytical method for the quantitative determination of 23 compounds in JMT was validated and evaluated using validation parameters such as linearity, recovery, precision, and accuracy [43]. For the calibration curves, working solutions of the mixed reference compounds were diluted to the appropriate concentrations and analyzed. Next, the calibration curve for each analyte was established by plotting the peak area ratios (analyte to IS) versus analyte concentrations. The lower limit of quantification (LLOQ) was defined as the lowest concentration of the standard curve that could be quantified with acceptable accuracy and precision. The limit of quantification (LOQ) was determined at an S/N ratio of 10. A recovery test was performed by adding three known concentrations (low, medium, and high) of each analyte to the JMT sample. The recovery was calculated using the following equation: recovery (%) = (found amount/spiked amount) × 100. To validate the precision of the analytical method, the mixed standard solution was measured six times and then evaluated using the CV value calculated from the measured concentration. The analysis of intra- and inter-day precision was performed with measurements for one day and three consecutive days for the three concentrations, respectively.

## 4. Conclusions

In this study, chemical profiling of JMT, a traditional herbal prescription, was performed using UHPLC-Q-Orbitrap-MS, and 23 marker compounds were identified. In addition, an analytical method for the quantification of the constituent compounds using UPLC-TQ-MS/MS was verified and developed. As a result, a total of 23 compounds were identified and compared to the authentic standard compounds. In the quantitative analysis performed using the developed MRM method, 23 components were simultaneously measured within 20 min, and all compounds were efficiently quantified. The qualitative and quantitative analysis methods established in this study enable efficient quality control of JMT and can also be utilized as important basic data for oriental medicine prescription. Furthermore, it can also be used in related research, such as the discovery of biologically active substances and analysis of efficacy.

## Figures and Tables

**Table 1:** Characterization of identified compounds of JMT by UHPLC-Q-Oribitrap-MS.

No.	Rt (min)	Formula	Adduct	Predicted (*m*/*z*)	Measured (*m*/*z*)	Error (ppm)	MS/MS Fragment	Identification
1	4.84	C_21_H_36_O_10_	[M + HCO_2_]^−^	493.2290	493.2290	−0.032	447.2233, 285.1705, 179.0553, 119.0335	Atractyloside A
2	5.19	C_25_H_41_NO_9_	[M + H]^+^	500.2854	500.2855	0.225	500.2856	Aconine
3	5.22	C_16_H_18_O_9_	[M + H]^+^	355.1024	355.1026	0.676	163.0390	Chlorogenic acid
4	5.17	C_15_H_14_O_6_	[M + H]^+^	291.0863	291.0863	0.503	165.0547, 139.0391, 123.0444,	Catechin
5	5.24	C_22_H_31_NO_3_	[M + H]^+^	358.2377	358.2377	0.173	358.2375, 340.2271	Napellonine
6	5.62	C_24_H_39_NO_7_	[M + H]^+^	454.2799	454.2800	0.151	454.2801, 436.2689	Fuziline
7	5.67	C_20_H_28_O_12_	[M + HCO_2_]^−^	505.1563	505.1563	0.061	165.0545	Paeonolide
8	5.64	C_15_H_14_O_6_	[M + H]^+^	291.0863	291.0863	0.083	207.0652, 165.0547, 139.0391, 123.0444	Epicatechin
9	5.85	C_24_H_39_NO_6_	[M + H]^+^	438.2850	438.2852	0.334	438.2851	Bullatine B
10	6.07	C_23_H_28_O_11_	[M + H]^+^	481.1704	481.1707	0.445	105.0340	Albiflorin
11	6.28	C_24_H_39_NO_5_	[M + H]^+^	422.2901	422.2900	−0.144	422.2901	Talatisamine
12	6.35	C_23_H_28_O_11_	[M + HCO_2_]^−^	525.1614	525.1611	−0.549	327.1086, 165.0546, 121.0280	Paeoniflorin
13	8.38	C_21_H_24_O_10_	[M − H]^−^	435.1297	435.1292	−1.054	273.0767, 169.0131	Phlorizin
14	10.90	C_30_H_32_O_12_	[M + HCO_2_]^−^	629.1876	629.1871	−0.806	121.0279	Benzoylpaeoniflorin
15	10.86	C_15_H_12_O_5_	[M + H]^+^	273.0758	273.0756	−0.690	273.0755, 153.0182, 147.0441	Naringenin
16	14.59	C_17_H_26_O_4_	[M + Na]^+^	317.1723	317.1723	−0.088	317.1724, 159.0417	6-Gingerol
17	15.54	C_15_H_2_0O_3_	[M + H]^+^	249.1485	249.1485	0.036	231.1382, 163.0754, 135.0442	Atractylenolide III
18	17.39	C_19_H_30_O_4_	[M+]^+^	322.2139	322.2140	0.306	304.2025, 205.0861, 150.0676, 137.0598	8-Gingerol
19	17.53	C_15_H_2_0O_2_	[M + H]^+^	233.1536	233.1537	0.590	233.1537, 215.1432, 187.1482, 151.0755	Atractylenolide II
20	18.22	C_30_H_48_O_4_	[M − H]^−^	471.3480	471.3478	−0.363	471.3474	Hederagenin
21	18.62	C_15_H_18_O_2_	[M + H]^+^	231.1380	231.1380	0.373	231.1380, 185.1326	Atractylenolide I
22	19.09	C_21_H_34_O_4_	[M+]^+^	350.2452	350.2453	0.401	332.2346, 150.0677, 137.0599	10-Gingerol
23	20.68	C_33_H_52_O_5_	[M + H]^+^	529.3888	529.3890	0.544	451.3570, 295.2426, 187.1481	Pachymic acid

**Table 2:** Regression equation, linear range, and lower limit of quantification (LLOQ) of the 23 compounds.

No.	Compound	Regression Equation	R^2^	Linear Range (ng/mL)	LLOQ * (ng/mL)
1	Atractyloside A	y = 0.0093x − 0.002551	0.9991	0.20–25.00	0.20
2	Aconine	y = 0.0961x − 0.000319	0.9991	0.02–3.13	0.02
3	Chlorogenic acid	y = 0.0289x − 0.000138	0.9990	0.05–6.25	0.05
4	Catechin	y = 0.0144x − 0.000465	0.9992	0.20–25.00	0.20
5	Napellonine	y = 1.1378x − 0.011170	0.9990	0.05–6.25	0.05
6	Fuziline	y = 0.3022x − 0.002625	0.9990	0.05–6.25	0.05
7	Paeonolide	y = 0.1614x + 0.000046	0.9990	0.02–3.13	0.02
8	Epicatechin	y = 0.0139x − 0.000102	0.9993	0.10–12.50	0.10
9	Bullatine B	y = 0.2041x − 0.003785	0.9991	0.10–12.50	0.10
10	Albiflorin	y = 0.0142x − 0.002176	0.9991	0.78–100.00	0.78
11	Talatisamine	y = 0.3650x − 0.003529	0.9991	0.05–6.25	0.05
12	Paeoniflorin	y = 0.1239x − 0.228416	0.9991	12.50–1600.00	12.50
13	Phlorizin	y = 0.1621x − 0.000523	0.9992	0.02–3.13	0.02
14	Benzoylpaeoniflorin	y = 0.1539x − 0.010071	0.9991	0.39–50.00	0.39
15	Naringenin	y = 0.0357x + 0.000024	0.9991	0.02–3.13	0.02
16	6-Gingerol	y = 0.0391x − 0.026660	0.9990	3.13–400.00	3.13
17	Atractylenolide III	y = 0.0350x − 0.000607	0.9991	0.10–12.50	0.10
18	8-Gingerol	y = 0.1039x − 0.001756	0.9990	0.10–12.50	0.10
19	Atractylenolide II	y = 0.0175x − 0.000245	0.9990	0.10–12.50	0.10
20	Hederagenin	y = 0.0036x − 0.000036	0.9990	0.05–6.25	0.05
21	Atractylenolide I	y = 0.0435x − 0.000087	0.9990	0.02–3.13	0.02
22	10-Gingerol	y = 0.0694x − 0.001277	0.9994	0.10–12.50	0.10
23	Pachymic acid	y = 0.0138x − 0.000105	0.9992	0.05–6.25	0.05

* LLOQ, lower limit of quantification.

**Table 3:** Recovery test for the assay of the 23 compounds in JMT.

No.	Compound	Spiked Concentration (ng/mL)	Measured Concentration (ng/mL)	Recovery (%)	RSD * (%)
1	Atractyloside A	14.48	15.58	107.60	0.82
		10.31	9.94	96.41	0.83
		8.23	7.16	87.00	2.03
2	Aconine	1.30	1.49	114.62	1.62
		0.78	0.86	110.26	2.86
		0.52	0.50	96.15	2.44
3	Chlorogenic acid	4.32	4.87	112.73	1.70
		3.28	3.48	106.10	1.62
		2.76	2.81	101.81	1.84
4	Catechin	17.96	18.49	102.95	1.94
		13.80	12.55	90.94	1.57
		11.71	10.14	86.59	1.27
5	Napellonine	3.27	3.57	109.17	3.13
		2.23	2.12	95.07	1.75
		1.71	1.50	87.72	2.36
6	Fuziline	3.39	3.62	106.78	3.11
		2.35	2.22	94.47	2.40
		1.83	1.61	87.98	2.16
7	Paeonolide	1.72	1.89	109.88	4.18
		1.20	1.15	95.83	1.90
		0.94	0.84	89.36	4.63
8	Epicatechin	7.68	7.53	98.05	3.76
		5.60	5.02	89.64	2.33
		4.56	4.08	89.47	1.17
9	Bullatine B	6.18	6.85	110.84	3.42
		4.09	4.00	97.80	1.43
		3.05	2.77	90.82	2.28
10	Albiflorin	73.91	77.19	104.44	1.12
		57.24	54.79	95.72	0.56
		48.91	44.42	90.82	0.85
11	Talatisamine	2.87	3.20	111.50	2.34
		1.83	1.89	103.28	2.02
		1.31	1.19	90.84	2.38
12	Paeoniflorin	1307.53	1322.90	101.18	0.82
		1040.86	944.34	90.73	0.88
		907.53	784.00	86.39	0.45
13	Phlorizin	1.25	1.43	114.40	2.78
		0.73	0.83	113.70	2.99
		0.47	0.46	97.87	5.80
14	Benzoylpaeoniflorin	31.01	32.74	105.58	1.74
		22.68	20.82	91.80	1.99
		18.51	16.16	87.30	0.66
15	Naringenin	1.17	1.32	112.82	1.87
		0.65	0.72	110.77	1.60
		0.39	0.41	105.13	4.89
16	6-Gingerol	245.96	263.74	107.23	0.72
		179.30	171.31	95.54	0.38
		145.96	128.42	87.98	0.61
17	Atractylenolide III	7.83	7.86	100.38	1.89
		5.75	5.46	94.96	1.03
		4.70	4.09	87.02	1.87
18	8-Gingerol	7.14	8.04	112.61	0.73
		5.06	5.29	104.55	1.03
		4.02	4.02	100.00	1.25
19	Atractylenolide II	9.42	9.17	97.35	1.18
		7.34	6.92	94.28	2.17
		6.30	5.43	86.19	1.02
20	Hederagenin	2.90	3.22	111.03	3.28
		1.86	1.88	101.08	1.78
		1.34	1.21	90.30	5.24
21	Atractylenolide I	1.48	1.61	108.78	2.00
		0.96	0.94	97.92	1.83
		0.70	0.64	91.43	3.06
22	10-Gingerol	9.37	9.23	98.51	0.51
		7.28	6.82	93.68	0.94
		6.24	5.51	88.30	0.85
23	Pachymic acid	3.14	3.43	109.24	3.88
		2.10	2.05	97.62	1.48
		1.58	1.43	90.51	3.60

* RSD, relative standard deviations.

**Table 4:** Precision and accuracy for the assay of the 23 compounds in JMT.

No.	Compound	Concentration (ng/mL)	Intraday		Interday	
			Precision (%)	Accuracy (%)	Precision (%)	Accuracy (%)
1	Atractyloside A	16.67	1.26	103.17	1.25	101.82
		8.33	2.11	100.98	0.20	101.20
		4.17	1.44	97.30	2.45	98.30
2	Aconine	2.08	1.56	110.74	2.89	107.22
		1.04	0.84	106.84	2.81	103.72
		0.52	1.65	102.39	1.01	101.27
3	Chlorogenic acid	4.17	2.16	101.73	8.00	94.34
		2.08	1.86	101.87	6.12	95.67
		1.04	1.98	97.69	7.12	90.27
4	Catechin	16.67	0.78	104.79	3.36	101.65
		8.33	0.82	101.26	2.81	98.90
		4.17	1.16	98.64	2.48	96.13
5	Napellonine	4.17	1.37	109.19	2.14	106.58
		2.08	1.36	104.06	2.09	101.62
		1.04	1.02	101.73	2.15	99.41
6	Fuziline	4.17	0.98	107.73	2.19	105.09
		2.08	0.73	103.55	2.18	101.01
		1.04	1.34	100.58	1.41	99.23
7	Paeonolide	2.08	3.13	98.49	3.11	99.18
		1.04	2.65	99.71	1.56	99.81
		0.52	6.50	97.87	4.95	96.42
8	Epicatechin	8.33	3.25	100.84	4.75	96.43
		4.17	2.22	98.22	2.54	96.14
		2.08	1.85	96.56	3.39	93.85
9	Bullatine B	8.33	0.71	108.76	2.66	105.53
		4.17	1.14	103.84	2.70	100.72
		2.08	1.33	100.04	1.16	99.15
10	Albiflorin	66.67	1.30	102.73	5.95	98.64
		33.33	0.99	99.52	6.86	95.92
		16.67	1.08	97.58	4.33	94.69
11	Talatisamine	4.17	1.53	108.12	1.32	106.56
		2.08	2.09	104.16	1.75	102.17
		1.04	1.22	101.78	1.57	100.82
12	Paeoniflorin	1066.67	1.81	100.72	2.94	101.35
		533.33	1.03	98.69	4.30	102.03
		266.67	0.95	95.43	2.83	98.54
13	Phlorizin	2.08	3.79	99.81	1.99	99.93
		1.04	1.64	95.61	2.17	98.07
		0.52	1.94	94.21	3.21	96.51
14	Benzoylpaeoniflorin	33.33	2.47	100.43	2.39	99.72
		16.67	1.84	97.41	1.90	98.23
		8.33	2.42	94.20	3.43	96.19
15	Naringenin	2.08	2.29	105.08	4.73	102.26
		1.04	2.10	102.13	4.62	98.89
		0.52	2.48	101.28	2.59	98.63
16	6-Gingerol	266.67	0.65	99.71	4.30	97.35
		133.33	0.33	95.21	5.28	92.24
		66.67	1.15	91.79	3.43	90.22
17	Atractylenolide III	8.33	1.11	101.56	3.91	98.88
		4.17	0.92	98.07	3.85	94.73
		2.08	1.49	97.14	3.50	93.75
18	8-Gingerol	8.33	0.85	102.44	5.66	97.99
		4.17	1.21	98.76	6.29	94.79
		2.08	0.75	96.25	5.28	92.38
19	Atractylenolide II	8.33	1.97	100.15	4.89	97.13
		4.17	1.75	98.61	3.19	96.14
		2.08	2.05	94.20	1.15	93.10
20	Hederagenin	4.17	2.88	104.96	4.16	100.81
		2.08	1.48	101.72	3.92	98.22
		1.04	3.81	100.53	3.57	97.25
21	Atractylenolide I	2.08	1.95	102.43	3.94	98.06
		1.04	1.28	97.79	2.32	95.25
		0.52	1.84	98.14	3.47	94.36
22	10-Gingerol	8.33	0.52	102.58	6.08	98.64
		4.17	1.55	98.58	6.46	94.72
		2.08	1.09	96.45	4.74	92.81
23	Pachymic acid	4.17	1.74	105.27	3.95	100.79
		2.08	2.25	100.70	3.29	97.11
		1.04	1.44	98.49	1.99	96.28

**Table 5:** Content of the 23 compounds in different batches JMT.

No.	Compound	JMT 1			JMT 2			JMT 3		
		Mean (mg/g)	SD	CV (%)	Mean (mg/g)	SD	CV (%)	Mean (mg/g)	SD	CV (%)
1	Atractyloside A	0.283	0.009	3.054	0.254	0.010	4.134	0.277	0.014	5.137
2	Aconine	0.013	0.000	1.952	0.013	0.000	2.073	0.013	0.000	3.655
3	Chlorogenic acid	0.076	0.004	4.962	0.060	0.001	1.650	0.073	0.003	4.021
4	Catechin	0.329	0.006	1.922	0.314	0.005	1.633	0.327	0.006	1.719
5	Napellonine	0.041	0.001	1.749	0.040	0.000	0.811	0.041	0.000	0.910
6	Fuziline	0.049	0.001	1.909	0.048	0.001	2.668	0.048	0.001	1.229
7	Paeonolide	0.065	0.002	3.814	0.057	0.003	4.654	0.065	0.002	2.862
8	Epicatechin	0.123	0.003	2.607	0.116	0.003	2.968	0.121	0.003	2.204
9	Bullatine B	0.072	0.001	1.713	0.070	0.001	2.089	0.070	0.001	1.708
10	Albiflorin	2.323	0.043	1.835	2.198	0.041	1.887	2.276	0.051	2.233
11	Talatisamine	0.028	0.000	0.938	0.027	0.001	2.019	0.027	0.000	1.482
12	Paeoniflorin	27.876	0.413	1.483	25.694	0.465	1.811	27.143	0.464	1.711
13	Phlorizin	0.007	0.000	6.391	0.007	0.000	5.582	0.007	0.000	3.499
14	Benzoylpaeoniflorin	0.452	0.004	0.871	0.426	0.009	2.090	0.436	0.007	1.662
15	Naringenin	0.004	0.000	2.783	0.004	0.000	6.109	0.004	0.000	3.783
16	6-Gingerol	4.919	0.056	1.139	4.877	0.082	1.674	4.724	0.067	1.419
17	Atractylenolide III	0.219	0.007	3.052	0.213	0.005	2.549	0.205	0.003	1.472
18	8-Gingerol	0.083	0.002	2.312	0.082	0.002	2.451	0.077	0.002	2.958
19	Atractylenolide II	0.306	0.008	2.621	0.296	0.009	3.099	0.280	0.007	2.398
20	Hederagenin	0.008	0.001	6.901	0.006	0.000	5.404	0.006	0.000	5.918
21	Atractylenolide I	0.008	0.000	2.244	0.008	0.000	1.064	0.008	0.000	5.637
22	10-Gingerol	0.029	0.001	3.462	0.028	0.001	2.735	0.027	0.000	1.604
23	Pachymic acid	0.008	0.000	1.677	0.008	0.000	1.924	0.008	0.000	2.193

**Table 6:** Composition of Jinmu-tang (JMT).

Scientific Name	Part Used	Ratio (%)
*Poria cocos* Wolf	sclerotium	24
*Paeonia lactiflora* Pallas	radix	24
*Zingiber officinale* Roscoe	rhizome	24
*Atractylodes japonica* Koidzumi	radix	16
*Aconitum carmichaeli* Debeaux	lateral radix	12

**Table 7:** MRM parameters of the 23 compounds and the internal standards.

No.	Compound	Rt (min)	Molecular Weight	Ion Mode	Precursor Ion (*m*/*z*)	Product Ion (*m*/*z*)	Collision Energy (V)
1	Atractyloside A	3.64	448.5	Negative	493.2	447.2	14
2	Aconine	3.92	499.6	Positive	500.3	450.3	40
3	Chlorogenic acid	3.94	354.3	Positive	355.0	163.0	10
4	Catechin	3.99	290.3	Positive	291.0	139.0	14
5	Napellonine	4.02	357.5	Positive	358.2	340.2	30
6	Fuziline	4.31	453.6	Positive	454.3	436.3	34
7	Paeonolide	4.38	460.4	Negative	505.2	165.0	26
8	Epicatechin	4.42	290.3	Positive	291.0	139.0	14
9	Bullatine B	4.52	437.6	Positive	438.3	420.3	30
10	Albiflorin	4.78	480.5	Positive	481.2	105.0	22
11	Talatisamine	4.96	421.6	Positive	422.3	390.2	30
12	Paeoniflorin	5.07	480.5	Negative	525.2	449.1	10
13	Phlorizin	7.08	436.4	Negative	435.1	273.1	10
14	Benzoylpaeoniflorin	9.56	584.6	Negative	629.1	553.2	10
15	Naringenin	9.61	272.3	Positive	273.0	153.0	26
16	6-Gingerol	13.27	294.4	Positive	277.1	177.1	10
17	Atractylenolide III	14.25	248.3	Positive	249.2	231.1	10
18	8-Gingerol	16.17	322.4	Positive	305.2	177.1	10
19	Atractylenolide II	16.34	232.3	Positive	233.1	187.1	14
20	Hederagenin	17.08	472.7	Positive	455.3	189.1	30
21	Atractylenolide I	17.45	230.3	Positive	231.0	185.1	18
22	10-Gingerol	17.93	350.5	Positive	333.2	177.0	10
23	Pachymic acid	19.49	528.8	Positive	511.3	451.3	18
IS1	Warfarin	13.70	307.1	Positive	309.0	163.0	14
IS2	Warfarin	13.71	307.1	Negative	307.0	250.0	22

## Data Availability

The data presented in this study are available on request from the corresponding author.
